# Multimodal analysis of ocular inflammation using the endotoxin-induced uveitis mouse model

**DOI:** 10.1242/dmm.022475

**Published:** 2016-04-01

**Authors:** Colin J. Chu, Peter J. Gardner, David A. Copland, Sidath E. Liyanage, Anai Gonzalez-Cordero, Sophia-Martha kleine Holthaus, Ulrich F. O. Luhmann, Alexander J. Smith, Robin R. Ali, Andrew D. Dick

**Affiliations:** 1 School of Clinical Sciences, University of Bristol, Bristol BS8 1TD, UK; 2 UCL Institute of Ophthalmology, 11-43 Bath Street, London EC1V 9EL, UK; 3 Roche Pharmaceutical Research and Early Development, Ophthalmology Discovery & Biomarkers, Roche Innovation Center Basel, F. Hoffmann-La Roche Ltd, Grenzacherstrasse 124, Basel 4070, Switzerland; 4 NIHR Biomedical Research Centre for Ophthalmology at Moorfields Eye Hospital and UCL Institute of Ophthalmology, London EC1V 9EL, UK

**Keywords:** EIU, Flow cytometry, OCT

## Abstract

Endotoxin-induced uveitis (EIU) in rodents is a model of acute Toll-like receptor 4 (TLR4)-mediated organ inflammation, and has been used to model human anterior uveitis, examine leukocyte trafficking and test novel anti-inflammatory therapeutics. Wider adoption has been limited by the requirement for manual, non-specific, cell-count scoring of histological sections from each eye as a measure of disease severity. Here, we describe a comprehensive and efficient technique that uses ocular dissection and multimodal tissue analysis. This allows matched disease scoring by multicolour flow cytometric analysis of the inflammatory infiltrate, protein analysis on ocular supernatants and qPCR on remnant tissues of the same eye. Dynamic changes in cell populations could be identified and mapped to chemokine and cytokine changes over the course of the model. To validate the technique, dose-responsive suppression of leukocyte infiltration by recombinant interleukin-10 was demonstrated, as well as selective suppression of the monocyte (CD11b+Ly6C+) infiltrate, in mice deficient for either *Ccl2* or *Ccr2*. Optical coherence tomography (OCT) was used for the first time in this model to allow *in vivo* imaging of infiltrating vitreous cells, and correlated with CD11b+Ly6G+ counts to provide another unique measure of cell populations in the ocular tissue. Multimodal tissue analysis of EIU is proposed as a new standard to improve and broaden the application of this model.

## Abstract (precis)

**Summary:** A refined platform approach for endotoxin-induced uveitis, using multimodal *ex vivo* and *in vivo* assessment, to improve quantification and phenotyping in this ocular model of compartmentalised tissue inflammation.

## INTRODUCTION

Acute self-resolving animal models of Toll-like receptor 4 (TLR4)-mediated inflammation such as peritonitis have been utilized widely to investigate the dynamics of leukocyte trafficking and the phenotypic changes of infiltrating cells that contribute to the resolution of inflammation (Stables et al., 2011). Although inflammation triggered by the TLR4 agonist lipopolysaccharide (LPS) has been extensively studied, we are still refining our understanding of the ensuing inflammatory response by different cells within tissues (Bowdish, 2014). Since it was first described in 1980, endotoxin-induced uveitis (EIU) in rats has been used extensively as a model of both central nervous system inflammation and acute ocular inflammation. Because the eye has multiple tissue compartments, interrogating inflammatory responses in this model offers the opportunity to investigate neural-specific tissue responses, as well as changes in the blood-retinal barrier and microenvironment (Rosenbaum et al., 1980).

Although not a direct correlate and model of ocular inflammatory disease in humans in respect to severity and chronicity, the model has traction because of the association between endotoxin-producing Gram-negative bacteria and HLA-B27-associated anterior uveitis, and offers an opportunity to elaborate our understanding of mechanisms of intraocular inflammation. The ability to induce EIU in mice provides a more rapid and cost-effective model for this disease, and the uptake of this model has been further increased owing to the new routes of disease induction, such as using intravitreal injection (Smith et al., 1998). Pathology is isolated to the confines of the eye and has a predictable monophasic peak, ideal for trafficking studies. Although EIU is predominantly used to emulate anterior chamber disease, distinct vitreous and retinal involvement also occurs in response to TLR4 challenge. As such, the model has also been employed for target identification and initial testing of novel drugs and therapeutic approaches for a wide variety of ocular disorders with an inflammatory component, such as age-related macular degeneration (Ildefonso et al., 2015).

However, the severity of EIU has previously only been scored using manual counts of infiltrating cells on paraffin sections across a limited sample of each eye. Such methods are inherently subjective, not amenable to high-throughput processing and limit the analysis of a single eye to disease scoring alone. This means that a separate, potentially asynchronous cohort would be needed to look at corresponding RNA, protein and tissue effects. Here, we describe a method of ocular dissection, dissociation and analysis that allows matched multimodal analysis upon each eye with EIU.

Furthermore, structural correlates can be achieved in this model using optical coherence tomography (OCT) as a non-invasive imaging technique that obtains near histological levels of detail based upon the scatter of reflected light from ocular tissues. This exploits the unique optical characteristics of the eye to visualize the effects of tissue inflammation. *In vivo* imaging using OCT has already been applied to score experimental autoimmune uveoretinitis (EAU) in mice (Chen et al., 2013; Chu et al., 2013). We describe a novel use of OCT to complement the multimodal analysis of EIU, by enabling *in vivo* measurement of infiltrating cells.

## RESULTS

### Quantifying infiltrating neutrophil numbers can be used to score disease severity in EIU

EIU can be initiated by either systemic or local administration of LPS; each route leads to different manifestations (Smith et al., 1998). Intravitreal administration was chosen because rapid and robust infiltration is obtained via direct challenge to the ocular environment. A technique for tissue dissection, dissociation and analysis by flow cytometry was developed, which additionally permits tissue and cellular supernatants to be obtained simultaneously from each eye for evaluation by quantitative real-time PCR (qPCR) or protein assay. Although EIU is often cited as a model of anterior chamber inflammation, the whole eye is affected and infiltrated, so aqueous humour, vitreous and retina were analysed together using our approach.

**Fig. 1.**
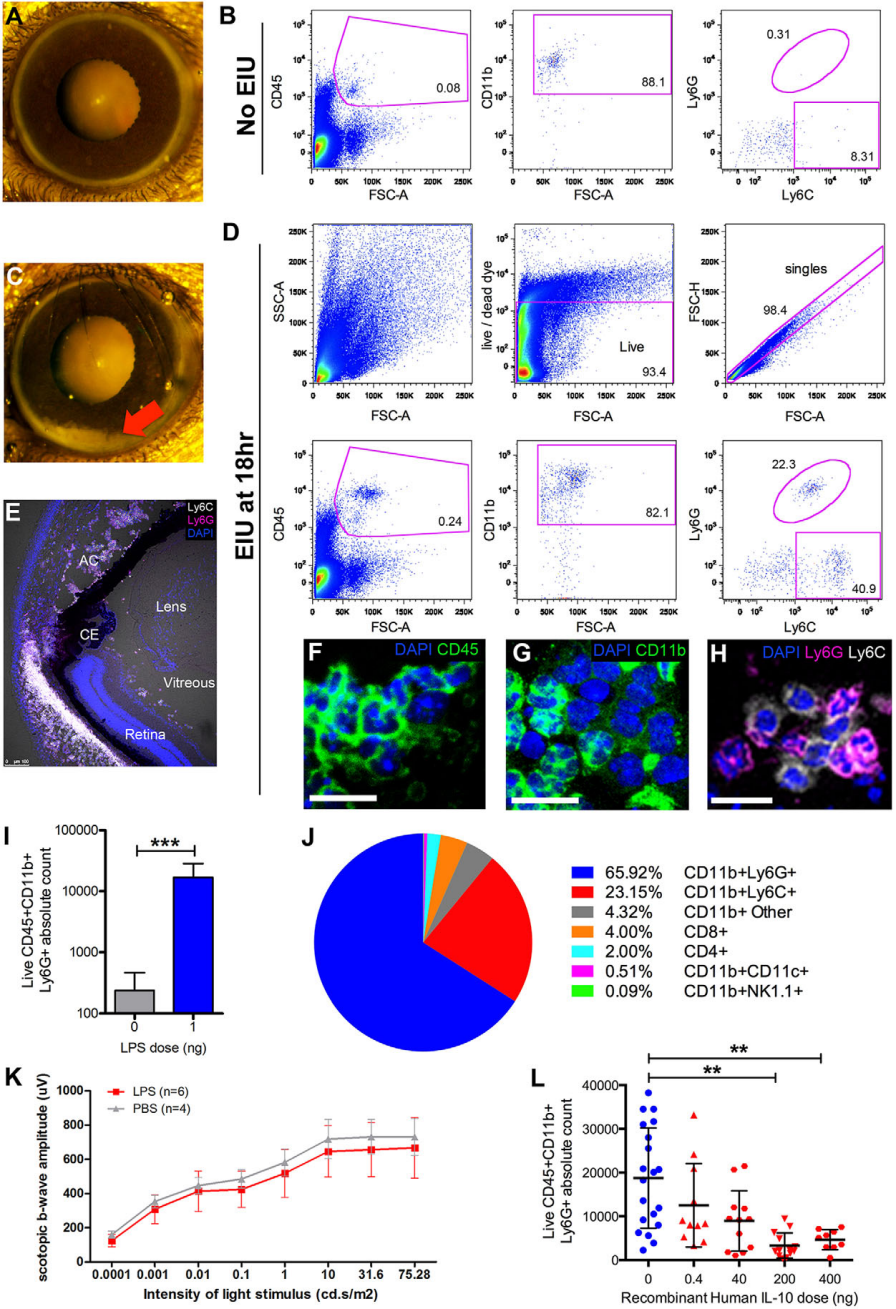
**Flow** **cytometry can quantify the leukocyte infiltrate during EIU and be used as a scoring system for identifying therapeutic effects.** In the absence of disease, (A) the anterior chamber is unremarkable and (B) negligible numbers of CD45+CD11b+Ly6G+ cells are detected by flow cytometry of whole ocular tissues. Numbers within the graphs in B indicate the percentage of each parent gate. At 18 h following intravitreal delivery of LPS, (C) marked ocular infiltration occurs throughout the eye, with leukocyte sediment (hypopyon) seen in the anterior chamber (indicated by the red arrow). (D) Representative example of the gating strategy to identify CD45+CD11b+Ly6G+ and Ly6C+ cells. (E) Immunohistochemistry targeting the same epitopes as the flow cytometry antibodies confirms the presence of leukocytes in the anterior chamber (AC) and vitreous, staining for (F) CD45 and (G) CD11b. CE, ciliary epithelium. (H) Ly6G+ cells (magenta) are polymorphonuclear, consistent with neutrophils, whereas Ly6C+ cells (white) appear mononuclear, consistent with monocytes. Scale bars: 20 μm. (I) Compared with untreated eyes, a statistically significant elevation in the number of neutrophils (CD45+CD11b+Ly6G+) can be seen during EIU and is proposed as a single measure score. Mann–Whitney *U*-test, *n*=20. (J) Using multicolour flow cytometry, the average cellular composition of the infiltrate in EIU was determined. Mean values from eight eyes undergoing standard processing. See Fig. S2 for the gating strategy. (K) Compared with PBS-injected control eyes, reduced retinal function is not seen by electroretinography 18 h after the induction of EIU, implying that the infiltrate is more critical than structure-function changes. Two-way ANOVA was used to compare the PBS and EIU cohorts. (L) To validate flow-cytometry-based scoring, reduced neutrophil infiltration was demonstrated using recombinant human IL-10, as previously published but using histological assessment (Rosenbaum and Angell, 1995). Each point represents one eye, with data pooled from four independent experiments, using 33 animals. One-way ANOVA. Mean±s.d. is shown in graphs. **P<0.01, ***P<0.001.

Control eyes were largely devoid of leukocytes apart from a population of CD45^low^CD11b^low^ cells that were Ly6G^neg^ and Ly6C^neg/low^, representing a retinal microglia phenotype (Fig. 1A,B). At 18 h following EIU induction, infiltrating leukocytes forming a sediment (hypopyon) in the anterior chamber were seen (Fig. 1C). Our flow cytometry strategy routinely excludes dead cells and doublets, before gating on CD45, then CD11b and dividing into Ly6G- and Ly6C-positive cells (Fig. 1D). We did not include the F4/80 marker because it is dispensable when identifying myeloid cells in the mouse (Rose et al., 2012). Immunohistochemistry using antibodies targeting the same epitopes as those used for flow cytometry confirmed the presence of cells expressing CD45, CD11b, Ly6G and Ly6C in the anterior chamber and vitreous (Fig. 1E-H). Of note, Ly6G+ cells were polymorphonuclear and Ly6C+ cells were mononuclear, consistent with their identity as neutrophils and monocytes, respectively (Fig. S1).

Compared with control eyes, flow cytometry could discern elevated numbers of neutrophils (CD45+CD11b+Ly6G+), which are acknowledged to be the principal effector cell (Smith et al., 1998) in EIU (Fig. 1I). Phenotyping the entire infiltrate at peak disease confirmed that this is the predominant population, followed by monocytes expressing Ly6C and only small numbers of T cells (Fig. 1J). Previous studies did not show marked structural changes by histology, but whether a more subtle pathology affects retinal function has not yet been investigated (Trittibach et al., 2008). Consequently, we performed electroretinography on eyes with EIU and in PBS-injected controls (Fig. 1K). No statistically significant difference was found, which supports the assessment of infiltration as the foremost criterion for EIU disease severity scoring. The absolute neutrophil cell count is proposed for this purpose.

To validate the use of this methodology as part of a multimodal assessment, we reproduced the suppression of EIU by simultaneous delivery of recombinant human IL-10, as had been previously published using histological scoring only (Rosenbaum and Angell, 1995). IL-10 titration exhibited a dose-responsive reduction in both mean and standard deviation (Fig. 1L). The control group illustrates the highly variable nature of the model, emphasizing the biological effect of therapeutic doses of IL-10, with this variation revealed by flow cytometry.

### Multimodal analysis allows dynamic changes in infiltration, cytokine levels and resident-tissue responses to be identified

**Fig. 2.**
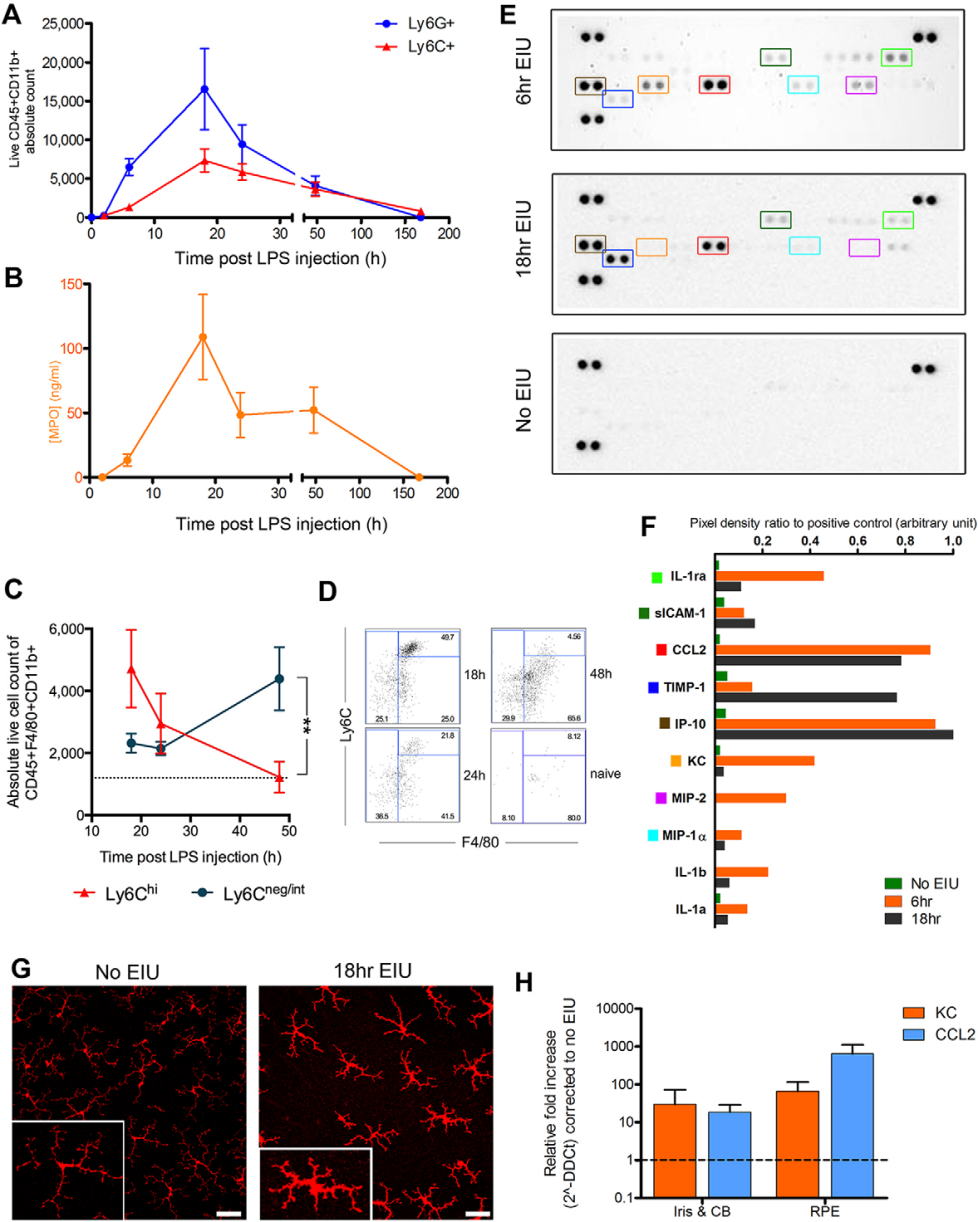
**Multimodal analysis characterises dynamic and plastic changes in infiltrating cells, protein levels and resident tissues during EIU.** Following intravitreal injection of 1 ng lipopolysaccharide (LPS), eyes were analysed by flow cytometry 2, 6, 18, 24, 48 and 168 h later. (A) The peak of CD45+CD11b+Ly6G+ (neutrophil) and CD45+CD11b+Ly6C+ (monocyte) cell numbers was at 18 h, with no infiltration seen at 2 h. By 7 days, cell numbers had returned to baseline. *n*=8 eyes per point, except at 6, 18 and 24 h, where *n*=22. (B) Myeloperoxidase (MPO) levels in supernatants from the same eyes used for flow cytometry reached peak level at 18 h. *n*=5 eyes per point. Connecting lines are for ease of interpretation and do not represent repeated measures upon the same eyes. Means±s.e.m. are shown. Single proteome profilers evaluated pooled supernatants from six dissociated eyes at each time point. (C) Flow cytometry analysis of F4/80 and Ly6C surface staining during the post-peak resolution phase of leukocyte infiltration, illustrating changes in the infiltrate composition with (D) representative cytometry plots. Data from two independent experiments; means±s.e.m. are shown. **P=0.003 at 48 h, Mann–Whitney *U*-test. *n*=8-11 eyes per point. (E) Developed images of R&D Mouse Cytokine Panel A membranes testing pooled supernatants from six eyes. Further representative examples are in Fig. S3. Key targets are outlined with coloured boxes, which correspond to the same coloured square markers listed in F. (F) Densitometry quantification of selected proteins from the representative membranes shown in E. (G) Retinal flat-mounts stained for *Iba-1* (red) show increased intensity alongside morphological changes in microglia populations, consistent with cell activation. Scale bars: 30 μm. Magnified example: inset, bottom left. In addition to infiltrating cells, resident cells might produce several cytokine/chemokines and as shown in H. (H) Residual tissues from the same group of eyes can be examined by qPCR and show elevated levels of KC and CCL2 relative to no-EIU controls. *n*=3 eyes per group; technical triplicates at 18 h post-induction; means±s.d. are shown. CB, ciliary body; RPE, retinal pigment epithelium.

Using ocular dissection and flow cytometry, peak numbers of neutrophils and monocytes were confirmed at 18 h post-induction, with the first infiltrating cells clearly detected by 6 h (Fig. 2A). At day 7, cell numbers had returned to baseline. Myeloperoxidase levels from matched supernatants at each time point confirmed that the peak levels correspond to peak cell numbers and likely reflect the inflammatory activation of many neutrophils (Fig. 2B). Further characterisation of phenotypic changes within the cellular infiltrate is possible using the myeloid surface marker F4/80 in conjunction with Ly6C (Fig. 2C,D). This revealed a shift within the ocular infiltrate from F4/80^+^Ly6c^hi^ inflammatory monocytes to F4/80^+^Ly6c^low^^-int^ macrophages during the resolution phase (18-48 h).

To look at combined cytokine and chemokine changes in the local microenvironment, matched supernatants from tissue dissociation were tested by proteome profiling across early and peak disease time points (Fig. 2E). These experiments highlighted early increases in pro-inflammatory cytokines such as KC (CXCL1), MIP-2 and IL-1β (Fig. 2F) associated with neutrophil recruitment. Similarly, CCL2 and IP-10 were elevated at both early and peak disease, and are likely contributors to monocyte recruitment; the successful detection of raised levels of these chemokines is also consistent with previous studies (Allensworth et al., 2011). Infiltrating cells will secrete these proteins but retinal pigment epithelium (RPE) or resident microglia can also produce them, and analysing supernatants with this technique captures the contribution of these two cell types. Microglial activation was confirmed by increased staining intensity of Iba-1 on retinal flat mounts and characteristic morphological changes from ramified to increased cell body forms (Fig. 2G).

The iris, ciliary body and RPE-choroid complex obtained during ocular dissection are not used for scoring, so our technique utilises them for further analysis. To isolate the contribution from other tissues, including the RPE, which is known to be involved in ocular immune processes, RNA can be obtained for eyes scored for EIU and analysed by qPCR. Increased transcript relative to eyes without EIU for both KC and CCL2 was seen, consistent with the protein profiler data (Fig. 2H). These targets are central to the two main cell populations involved in EIU, with KC being the primary chemokine for neutrophils, whereas CCL2 is crucial for monocyte recruitment. Tissues were washed in PBS prior to RNA extraction, but it cannot be guaranteed that infiltrating cells were completely removed. These forms of matched analysis are only possible owing to parallel multimodal assessment and would not be available with conventional histological scoring.

### Multimodal EIU analysis can identify differential effects upon cell subsets, as demonstrated with *Ccl2-* and *Ccr2*-deficient mice

**Fig. 3.**
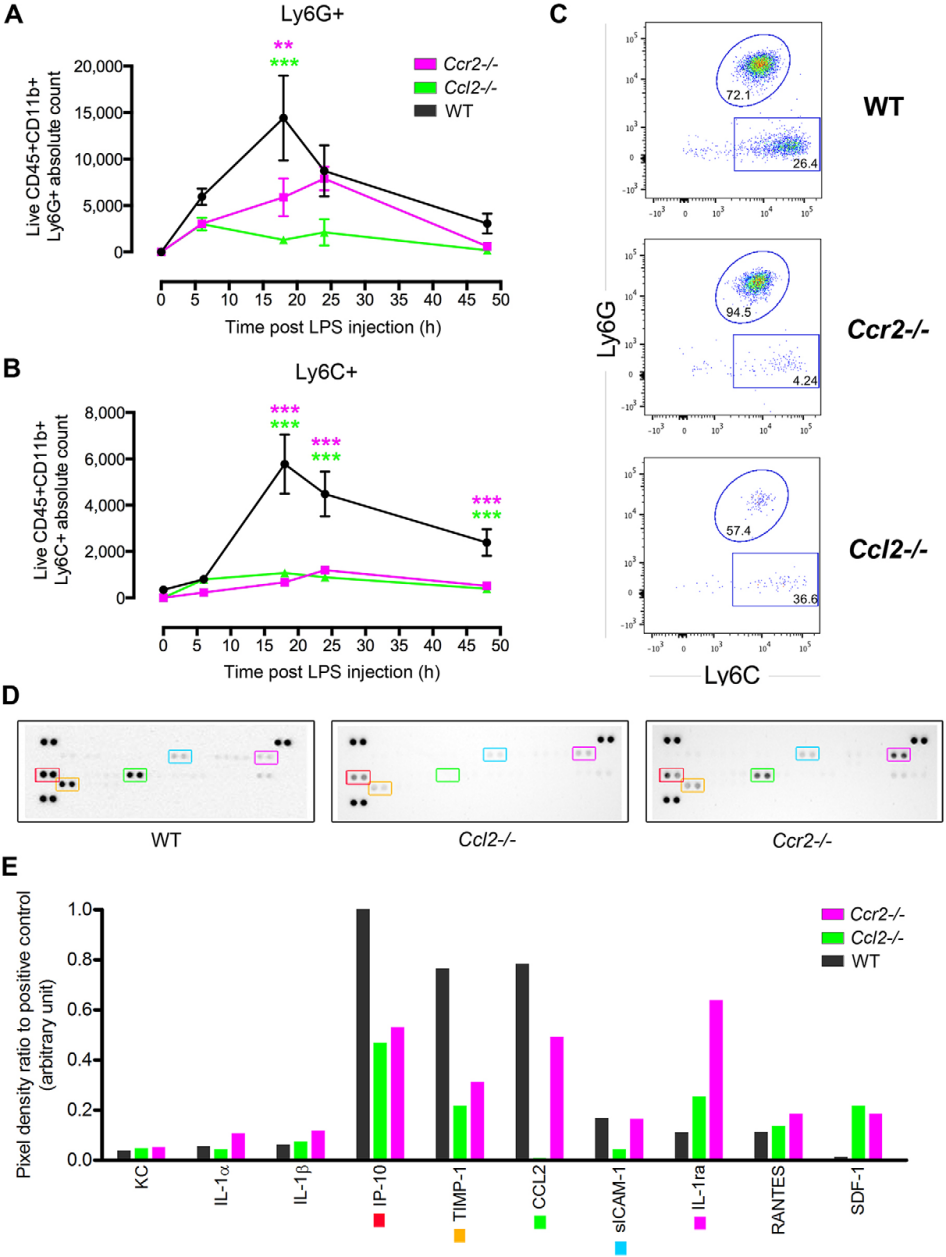
**Either** ***Ccl2* or *Ccr2* deficiency prevents monocyte infiltration in EIU but attenuates neutrophil numbers at peak disease only.** Using *Ccl2-* or *Ccr2*-deficient mice, the differential effects upon Ly6G+ and Ly6C+ populations can be identified within the same sample by flow cytometry. (A) Over the course of 48 h following EIU induction, compared with wild-type (WT) mice, lower numbers of CD11b+Ly6G+ cells are seen in *Ccl2*^−/−^ and *Ccr2*^−/−^ mice, but this only reaches statistical significance at 18 h. This is despite (B) minimal numbers of CD11b+Ly6C+ cells in both *Ccl2*^−/−^ and *Ccr2*^−/−^ mice with EIU at all time points. *n*=4-16 eyes per time point from two independent experiments. Two-way ANOVA and Tukey multiple comparison test: **P<0.01, ***P<0.001. Means±s.e.m. are shown. (C) Representative flow cytometry plots at 24 h post-EIU induction, illustrating comparable numbers of Ly6G+ cells but reduced counts of Ly6C+ cells. The numbers within the graphs indicate the percentage of each parent gate. (D) Supernatants of six dissociated eyes at 18 h of EIU were pooled and analysed by R&D Mouse Cytokine Panel A proteome profiler. Key targets are outlined with coloured boxes, which correspond to targets of the same colour marker in E. (E) Densitometric quantification of selected proteins.

The role of CCR2 in monocyte migration into inflamed tissues might depend on the stimuli and tissue; however, the presence and number of circulating blood-borne inflammatory monocytes is controlled by the CCR2-CCL2 axis, whereby Ly6C^hi^ monocytes express CCR2, allowing their exit from the bone marrow into the blood circulation (Serbina and Pamer, 2006). One role of CCR2 then is to maintain a pool of circulating inflammatory monocytes and facilitate release of replenishing monocytes when required from the bone marrow that can then respond to inflammatory stimuli. Additionally, CCR2 might play a role in regulating monocyte trafficking into the eye because, whereas retinal expression of CCL2 is very low in healthy young adult mice, it increases during acute inflammation (Raoul et al., 2010) and is also required for correct functioning of the para-inflammatory response responsible for protecting the retina against age-related degeneration (Chen et al., 2011). Given the detection of high levels of CCL2 during EIU, the effect of germline *Ccl2* or *Ccr2* deletion and the ability of multimodal assessment to detect differences in inflammation was tested by comparing wild-type mice to two knockout lines. Measured simultaneously on the same eyes, mean neutrophil numbers were only moderately lower in knockout eyes (Fig. 3A), but with minimal numbers of monocytes detected in the eye at any stage (Fig. 3B,C). This is consistent with the dominant role of the CCR2-CCL2 axis for the release of monocytes from the bone marrow and recruitment into inflamed tissue (Boring et al., 1997). Protein profiling of matched supernatants demonstrated a generalised reduction in IP-10 and TIMP-1, with a large increase in IL-1ra in *Ccr2*-deficient mice and a decrease in sICAM-1 in *Ccl2*-deficient mice only (Fig. 3D,E). Correlating these environmental protein alterations with cell infiltrate changes is only possible using our technique and provides data that can provide deeper insight into biological mechanisms.

### OCT correlates with flow cytometry scores and can be used to assess EIU *in vivo*

**Fig. 4.**
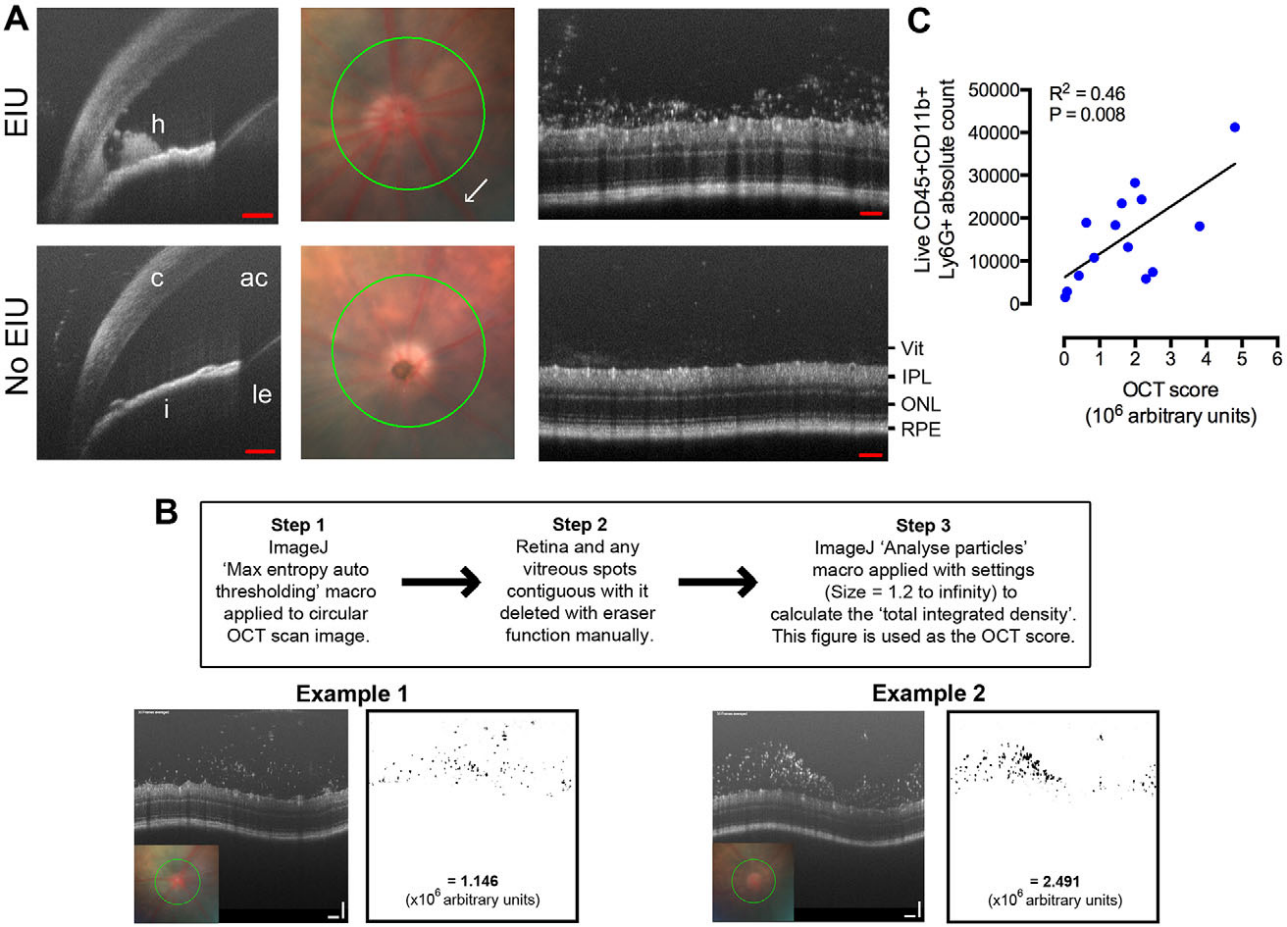
***In vivo* EIU assessment using OCT correlates with flow-cytometry-based counts.** 18 h after lipopolysaccharide (LPS) administration, eyes were imaged by OCT. (A) Hypopyon and static aggregations of cells in the anterior chamber (AC) could be detected. Circular scans (green) were centred on the optic disc using fundus images and the vitreous infiltrate scored. Vascular dilatation can be seen in EIU eyes (white arrow and Fig. S4). h, hypopyon; c, cornea; i, iris; ac, anterior chamber; le, lens; vit, vitreous cavity; IPL, inner plexiform layer; ONL, outer nuclear layer; RPE, retinal pigment epithelium. Scale bars: 100 μm. (B) Method and worked examples of OCT score calculation using ImageJ software (Rasband, W. S., ImageJ, US National Institutes of Health, Bethesda, MD, USA, imagej.nih.gov/ij/, 1997-2012). (C) After imaging, eyes were dissected, processed by flow cytometry and a statistically significant correlation to their *in vivo* OCT scores were demonstrated. *R*^2^=0.46, *P*=0.008, *n*=14, Pearson correlation. Linear regression is shown.

Using OCT, the presence or absence of disease was discernible *in vivo* by visualising cellular infiltration in the anterior and posterior chambers of the eye (Fig. 4A). Vascular dilation and retinal thickening, due to inflammation-induced tissue oedema, were visible. Cells such as those forming aggregates in the anterior chamber or entrapped within the vitreous gel were visualised. Although cells percolate in the anterior chamber in human anterior uveitis, they could not be visualised by OCT in the mouse with our system owing to the slow speed of image acquisition and cell movement by aqueous currents. Newer OCT machines specially adapted for fast imaging have been able to detect anterior chamber (AC) cells in humans, but are currently unavailable for animal work (Rose-Nussbaumer et al., 2015).

The vitreous around the optic disc was chosen as a reference point for imaging posterior chamber cells, as this is a known site for early infiltration. The scan of each eye was processed as described in Fig. 4B to obtain an OCT-based score. The corresponding eyes were dissected and assessed by flow cytometry, and the total neutrophil count was shown to correlate statistically significantly with this score (Fig. 4C). This form of *in vivo* imaging could therefore be used alone for rapid screening or repeated assessment upon the same eye, or combined to complement subsequent *ex vivo* multimodal analysis.

## DISCUSSION

This article presents a unique multimodal platform for examining EIU. By performing ocular dissection as described, multiple datasets can be obtained from the same sample, providing a powerful means to dissect the interplay between the cellular infiltrate and tissue. Flow cytometry used to accurately quantify and phenotype the infiltrate, multiplexed protein quantification on supernatants and RNA transcript assessment of resident cells can all be used to examine changes in the local immune microenvironment. Even if used alone, flow cytometry to score EIU is rapid, more quantitative and more amenable to high-throughput processing than histology. This is of clear benefit in the screening of new ocular therapeutics (Table 1 summarises the differences between the two techniques) because obtaining matched multimodal data for each eye can more robustly identify disease suppression and provide an indication of key changes to the microenvironment.

**Table 1.**
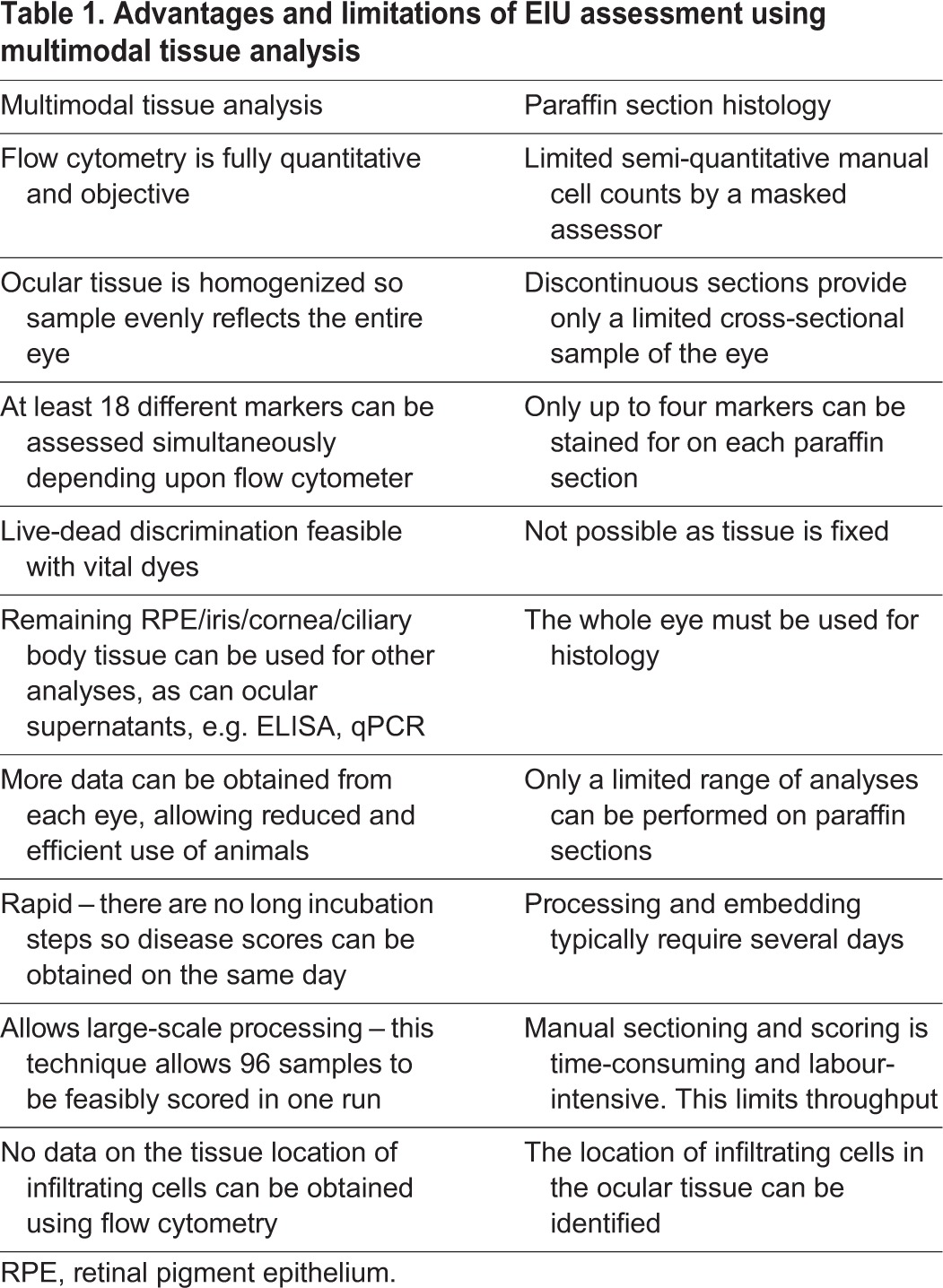
**Advantages and limitations of EIU assessment using multimodal tissue analysis**

We describe for first the time the use of multicolour flow cytometric counts for scoring EIU based on cell-surface phenotype. Infiltrate quantification based on histology can provide estimates of total cell numbers in the eye using calculations based on assumptions of homogeneity of cells/area/section, and is limited to several immunohistochemical stains at a time. As this data shows, we are able to accurately quantify multiple cell types measured on the same sample of the same eye. In addition, our new approach to the assessment of EIU has provided additional insights into changes in cellular constituents throughout the course of disease. This improved characterisation should widen the scope of its use and refine both the data available and the scientific questions that can be answered.

A feature that is consistent with other models of self-resolving, sterile inflammation is the change in populations of monocytes/macrophages infiltrating tissues over the course of the model (Crane et al., 2014; Newson et al., 2014). Our combination of cell surface phenotyping and accurate quantification by flow cytometry has revealed similar population dynamics in the eye, revealing that non-inflammatory regulatory cells dominate in the post-peak phase. In the future, further expansion of the cell surface markers used will further reveal important cellular dynamics involved in the resolution of inflammation, and which can be therapeutically manipulated.

Electroretinography is commonly used to measure gross changes in retinal function in mice and has been used to detect structural damage caused by autoimmune inflammatory infiltrate in EAU (Yazid et al., 2015). We found no marked loss of retinal function, despite retinal leukocyte infiltration. This data is arguably not an unexpected finding because the inflammation is not an antigen-driven process and resolves, but it poses questions about important regulatory mechanisms controlling myeloid responses within ocular tissues in the absence of active infection or antigen-specific autoimmunity. Importantly, it has been shown in EAU that the presence of leukocytes does not necessarily correlate with tissue damage because therapeutic TNF inhibition can alter the activation status of infiltrating cells (Dick et al., 1996). Our analysis using OCT does reveal microvascular structural changes in the retina associated with inflammation, challenging the paradigm that EIU is purely a model of cell trafficking (Smith et al., 1998). Although not performed here, rapid multiple measurements on the same eye, using OCT, is possible over time to examine trafficking kinetics. The benefit of this approach is a potential reduction in the number of animals required for experiments, in line with the 3Rs (replacement, reduction and refinement), and more accurate assessment of disease because variation is reduced by using the same eyes repeatedly. Anterior segment spectral domain OCT (SD-OCT) has been performed following topical application of LPS (Downie et al., 2014), but we further these observations by characterising EIU and examining the posterior chamber in correlation to the inflammatory infiltrate. In the future, the use of OCT assessment of EIU will also inform and facilitate the comparison of this model with the assessment of human ocular inflammatory disease, refining the application of findings in EIU to human therapy (Rose-Nussbaumer et al., 2015).

Demonstrating the potential and utility of this multi-modal platform, our examination of EIU in *Ccr2-* and *Ccl2*-deficient mice reveals that the neutrophil response is not wholly independent of *Ccl2*, suggesting perhaps that the induction of some neutrophil chemoattractants are downstream of *Ccl2* signalling. Indeed, we highlight subtle differences between the presence of *Ccr2* and *Ccl2* regarding the expression of the chemokines TIMP-1, sICAM-1 and IL1ra, opening opportunities for further investigation.

Although EIU is an acute, self-resolving disease model restricted to myeloid infiltration, arguably its utility has been limited by the constraint of its analysis by histological assessment alone. Other models, such as T-cell-driven EAU, provide a closer parallel of human disease, but are generally more expensive and slower, and so less conducive to early proof-of-concept therapeutic studies or rapid screening. Alternative models of acute inflammation, such as zymosan peritonitis or caecal ligation and puncture, cause more severe distress to the animals, and *in vivo* measurement of the trafficking process is difficult to follow. EIU shares pathophysiological similarities but, with the use of OCT, could allow for repeated *in vivo* assessment.

Application of our approach for routine EIU assessment can improve the efficiency, value and uptake of the model, and expand its utility into fields such as trafficking, the interplay between resident and infiltrating cells, and mechanisms of inflammation resolution (Newson et al., 2014).

## MATERIALS AND METHODS

### Mice and EIU induction

Unless stated otherwise, all mice used were female C57BL/6J strain (Harlan, UK), brought into the animal facility at 6 weeks of age and maintained on the open shelf with food and water *ad libitum* a week prior to procedure. Breeding pairs of C57BL/6 *Ccr2*^−/−^ (B6.129S4-Ccr2tm1Ifc/J) mice were a gift from Derek Gilroy, UCL and C57BL/6 *Ccl2*^−/−^ mice from Kath Else, University of Manchester. Knockout mouse lines were bred and kept homozygous for the relevant chemokine loci, and experiments performed on mice aged between 2 and 6 months of age. Mouse strains were tested to be free of the *Crb1*RD^8/RD8^ mutation (Luhmann et al., 2012). All procedures conformed to the Association for Research in Ophthalmology (ARVO) statement for use of animals in ophthalmic and vision research.

Under anaesthesia, a 34-gauge needle (Hamilton, Switzerland) was passed perpendicular to the sclera, 0.6 mm from the corneal limbus into the vitreous cavity. 2 μl PBS containing 1 ng LPS from *E.coli* 055:B5 (Sigma-Aldrich, UK) or recombinant human IL-10 (Invitrogen Life Technologies, UK) was injected.

### Electroretinography

Scotopic examinations were performed under single-flash recording using increasing light intensities of 0.0001, 0.001, 0.01, 0.1, 1, 10, 31.6 and 75.28 cd.s/m^2^ with adaptation times of 1, 8, 15, 20, 90, 100 and 120 s between each increasingly bright flash, respectively. a- and b-wave amplitudes were detected and analysed using the Espion system (Diagnosys LLC, Cambridge, UK).

### *In vivo* imaging and scoring

Following anaesthesia and dilation with 1% tropicamide, mice were imaged using an SL-16 slit-lamp (Keeler, Windsor, UK) for anterior segment photographs, and a Micron IV camera (Phoenix Research Laboratories, USA) for OCT and fundal images. EIU scoring by using vitreous cell densitometry was performed on circular retinal OCT images centred on the disc using ImageJ software.

### Ocular dissection and flow cytometry

The following describes the standard technique used throughout the paper, referred to as ‘standard preparation’, which pools aqueous humour, dissociated retina and vitreous for each eye. All supernatants used for analysis were derived from this combination of tissues. The matching RPE-choroid complex was not pooled but separately analysed only where specifically indicated. Each eye was dissected in 100 μl PBS on a Petri dish. Surrounding extraocular muscle, fat and optic nerve were removed. A puncture was made at the corneal limbus with a 25 G needle. Curved Vanna scissors were used to extend this cut around the circumference of the eye. The cornea and iris were removed with forceps and the aqueous washed into the PBS. The lens was extracted anteriorly with two pairs of forceps. The retina and encapsulated vitreous typically lifts free along the subretinal space, remaining attached to the lens capsule. The residual sclera-choroid-RPE and ciliary body complex was removed to another Petri dish, dissected into constituents, washed twice in PBS and snap-frozen in liquid nitrogen for subsequent RNA extraction separately.

The lens was discarded, with vitreous and retina washed within the 100 μl PBS containing the aqueous humour, before transfer into a 1.5 ml microcentrifuge tube, mechanical dissociation by rapping the tube across a 12-well Eppendorf rack ten times and the resulting solution was pipetted into a 96-well 60-μm cell strainer plate (Merck Millipore, Watford, UK). The tube was washed with a further 100 μl PBS. This was centrifuged at 300 ***g*** for 5 min, the supernatants removed for separate analysis and the remaining cell pellet transferred into a 96-well V-bottom plate. Cells were incubated with fixable viability dye eFluor780 (eBioscience, San Diego, USA) before Fc-block (BD Biosciences, Oxford, UK) and primary conjugated-antibody staining. Antibodies were used at a 1 in 100 dilution and were from eBioscience [Ly6C (HK1.4, 12-5932), F4/80 (BM8, 48-4801), CD11b (M1/70, 25-0112), CD8 (H35-17.2, 48-0083)], BioLegend [Ly6G (1A8, 127614), CD45 (30-F11, 103133)] or BD Biosciences [CD4 (L3T4, 557308), CD11c (HL3, 557400), NK1.1 (PK136, 561117)]. LSRII and Fortessa X-20 machines (BD Biosciences) were used for acquisition with fixed medium flow rates for 45 s and analysis performed on FlowJo (Treestar, USA). Compensation was performed using OneComp eBeads (eBioscience) and ArC amine reactive beads (Invitrogen Life Sciences, UK). Fluorescence minus one (FMO) controls were used to determine gate position. Eight two-fold serial dilutions of a known concentration of Sphero AccuCount blank polystyrene beads (Spherotech, USA) were acquired identically to construct a standard curve and estimate absolute cell numbers.

### Immunohistochemistry

Eyes were frozen in OCT medium (Fisher Scientific, UK) and 12-μm cryosections stained with anti-mouse Ly6C-PE (HK1.4, eBioscience), anti-mouse Ly6G-APC (1A8, BioLegend), anti-mouse CD11b (M1/70, BD Biosciences) and anti-CD45 (30-F11, eBioscience). Retinal flat-mounts were dissected and placed into 1% BSA/3% Triton X-100/ 5% normal goat serum and incubated overnight at 4°C with 1:200 anti-mouse Iba-1 (Wako, Osaka, Japan). Retinas were incubated with Alexa-Fluor-594 (Invitrogen Molecular Probes) secondary antibody for 4 h, mounted and imaged with a Leica DM5500Q confocal microscope.

### Quantitative PCR

cDNA was produced using the QuantiTect Reverse Transcription Kit, following RNA extraction with an RNeasy Mini Kit (Qiagen, USA). qPCR was performed using the Universal ProbeLibrary system-designed primers, master mix (Roche diagnostics, UK) and an Applied Biosciences 7900HT thermal cycler. Primers used included mouse KC (5′-AGACTCCAGCCACACTCCAA-3′ and 5′-TGACAGCGCAGCTCATTG-3′) and CCL2 (5′-CATCCACGTGTTGGCTCA-3′ and 5′-GATCATCTTGCTGGTGAATGAGT-3′). Samples were run in triplicate and analysed using the 2^−ΔΔCT^ method relative to β-actin.

### Protein analysis

Pooled supernatants (derived from the standard dissection of vitreous, retina and aqueous humour) from six eyes were used for each proteome profiler (R&D Systems, USA) and developed according to the manufacturer's guidelines, imaged with a Nikon LAS4000 CCD imager and densitometry performed with ImageJ. Membranes were normalised to each other using all six positive control spots and a background reading. Mouse myeloperoxidase ELISA (Hycult Biotech, Netherlands) was used, according to the manufacturer's instructions, with single-eye supernatants.

### Statistical analysis

Depending on the nature of the data, Mann–Whitney *U*-tests, Pearson correlation or one- and two-way ANOVA were performed as specified in the figure legends.
